# Transcriptional Regulation of T-Cell Lipid Metabolism: Implications for Plasma Membrane Lipid Rafts and T-Cell Function

**DOI:** 10.3389/fimmu.2017.01636

**Published:** 2017-11-24

**Authors:** George A. Robinson, Kirsty E. Waddington, Ines Pineda-Torra, Elizabeth C. Jury

**Affiliations:** ^1^Centre of Rheumatology, Division of Medicine, University College London, London, United Kingdom; ^2^Clinical Pharmacology, Division of Medicine, University College London, London, United Kingdom

**Keywords:** T-cells, lipid rafts, cholesterol, glycosphingolipids, fatty acids, nuclear receptors, autoimmunity, gender

## Abstract

It is well established that cholesterol and glycosphingolipids are enriched in the plasma membrane (PM) and form signaling platforms called lipid rafts, essential for T-cell activation and function. Moreover, changes in PM lipid composition affect the biophysical properties of lipid rafts and have a role in defining functional T-cell phenotypes. Here, we review the role of transcriptional regulators of lipid metabolism including liver X receptors α/β, peroxisome proliferator-activated receptor γ, estrogen receptors α/β (ERα/β), and sterol regulatory element-binding proteins in T-cells. These receptors lie at the interface between lipid metabolism and immune cell function and are endogenously activated by lipids and/or hormones. Importantly, they regulate cellular cholesterol, fatty acid, glycosphingolipid, and phospholipid levels but are also known to modulate a broad spectrum of immune responses. The current evidence supporting a role for lipid metabolism pathways in controlling immune cell activation by influencing PM lipid raft composition in health and disease, and the potential for targeting lipid biosynthesis pathways to control unwanted T-cell activation in autoimmunity is reviewed.

## Introduction

CD4^+^ T-cells play a central role in the adaptive immune system. Upon activation, they proliferate, traffic to inflamed sites, and acquire functions that mediate the immune response against infection and malignancy (1). These processes have significant metabolic demands and understanding how metabolites (including glucose, amino acids, and cholesterol) are modulated to meet these increased energetic demands is an urgent challenge (1). The majority of current studies refer to changes in intracellular metabolites and how they affect T-cell function. In this review, we will focus on the role of cellular lipid metabolism in the regulation of plasma membrane (PM) lipid composition and the importance of this to T-cell function—a mechanism which has only just begun to be explored (2, 3).

## T-Cell PM and Lipid Rafts

The T-cell PM provides a flexible interface where signals generated by cell surface receptors lead to functional outcomes, including activation, proliferation, and cytokine production. Lipids and proteins are both essential PM constituents, but while PM proteins have been widely studied, there is a gap in our knowledge about the fundamental role and regulation of lipid PM components (4). This gap impedes our understanding of how PM lipids influence immune cell function and how they could be targeted or manipulated therapeutically.

Cholesterol and glycosphingolipids (GSLs) are particularly enriched in the PM and form signaling platforms known as lipid rafts. Signaling molecules accumulate at high density in lipid rafts and they are essential for immune cell activation and function (5, 6).

Cholesterol helps to maintain lipid raft structure; the amount of cholesterol, cholesterol intermediates such as lanosterol, or oxidized cholesterol in the PM can alter lipid raft stability and affect cell function by modifying the lateral mobility of membrane receptors and signaling molecules (7–11). More specifically in T-cells, PM cholesterol has been shown to mediate T-cell receptor (TCR) clustering, inhibit spontaneous TCR activation and reduce TCR mobility in the membrane (12–14). Similarly, GSLs influence T-cell functions including TCR-mediated signaling and responsiveness to cytokine stimulation (15–18), apoptosis, and recycling/endocytosis of membrane signaling and receptor molecules (19). Changes in lipid composition affect the biophysical properties of PM lipid rafts (20). Studies also show that distinct PM lipid profiles (GSL and cholesterol content) are associated with well-defined T helper (Th) cell subsets (Th1, Th2, and Th17) (15, 17, 18, 21, 22), supporting a role for PM lipid composition in defining functional T-cell phenotypes (23). Interestingly, changes in PM lipid order, measured using the fluorescent membrane probe di-4-ANEPPDHQ, can dictate the response of T-cells to TCR stimulation. T-cells with high PM order form more stable immune synapses, proliferate robustly and favor a Th-2 phenotype whereas T-cells with lower levels of PM order form more unstable immune synapses, have reduced proliferative capacity and produce more proinflammatory cytokines. For instance, reducing PM order with the oxysterol 7-ketocholesterol is alone sufficient to alter the functional phenotype of T-cells (9).

These advances in understanding the link between PM lipids and T-cell function are supported by state-of-the-art microscopy techniques including super-resolution fluorescence microscopy that have revolutionized the visualization of PM lipids and membrane order (24–28). The increasing evidence describing defects in T-cell PM lipid rafts associated with abnormal T-cell function in autoimmunity makes this an attractive therapeutic area (29, 30).

## Transcriptional Regulators of Lipid Metabolism and Lipid Rafts

### Liver X Receptors (LXRs)

**Figure 1:**
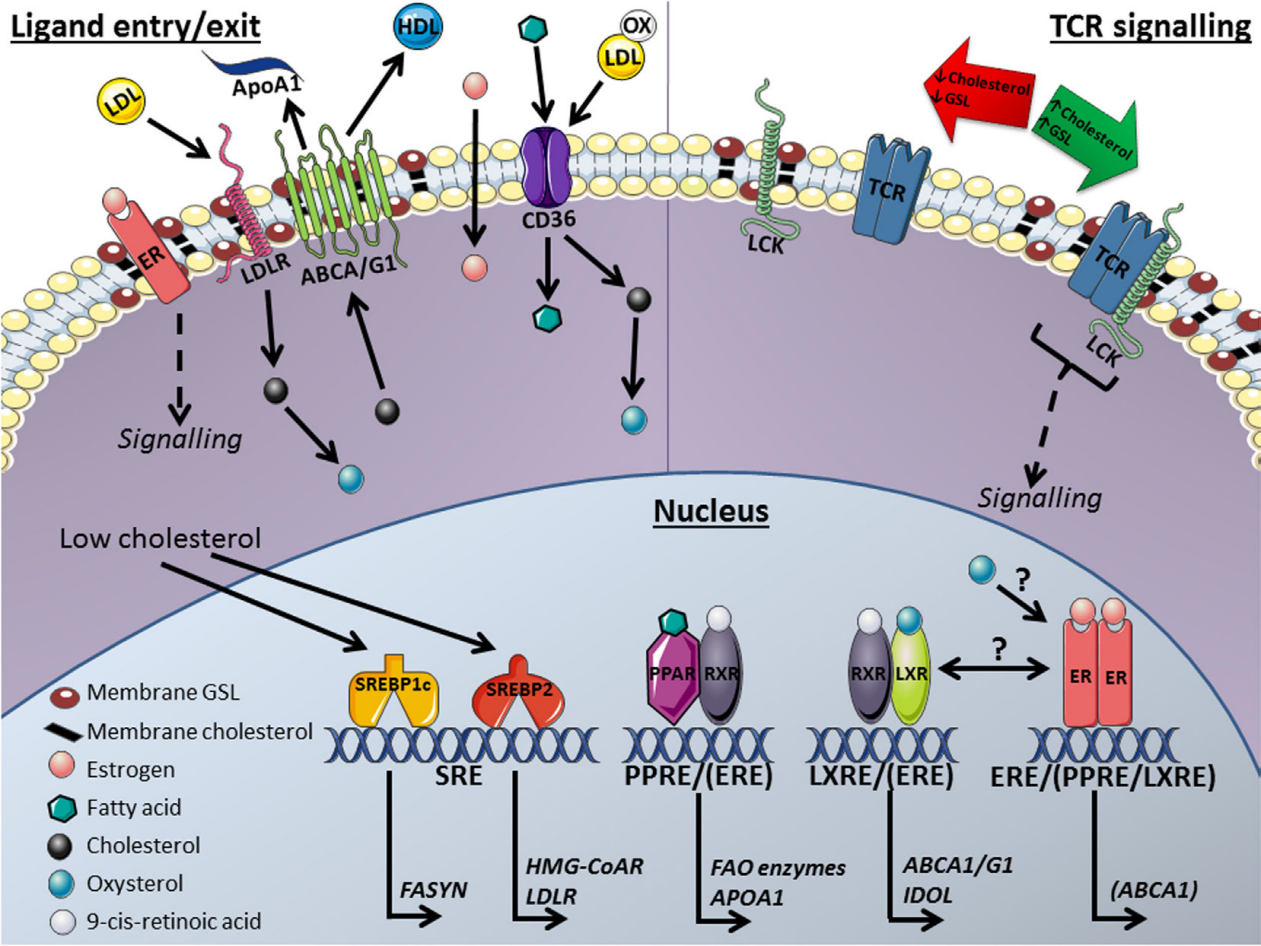
Mechanisms for the transcriptional regulation of lipid metabolism proposed to influence plasma membrane lipid rafts and T-cell function: This model includes key nuclear and membrane receptors and transcription factors that are affected by and influence (membrane) lipid metabolism and T-cell activation. Ligand entry/exit: membrane receptors; arrows indicate direction of lipid molecule transport in and out of the cell. ATP-binding cassette transporters (ABCA1/G1) efflux cholesterol from the cell to high-density lipoprotein (HDL) or lipid poor apolipoprotein A1 (apoA1) molecules. Cholesterol is imported into the cell through low-density lipoprotein receptors (LDLRs) and CD36 transporters from low-density lipoprotein (LDL) molecules. Fatty acids enter the cell with binding proteins or *via* CD36 transport. Nucleus: sterol regulatory element-binding proteins (SREBPs) regulate the transcription of fatty acid synthase (FASYN), LDL-receptor (LDLR) and 3-hydroxy-3-methyl-glutaryl-coenzyme A reductase (HMG-CoAR) through sterol regulatory elements (SREs) in response to low cholesterol levels. Peroxisome proliferator-activated receptor (PPAR) stimulation by fatty acids induces the transcription of fatty acid oxidase (FAO) enzymes and apoA1 at PPAR response elements (PPREs) following dimerization with the retinoid X receptor (RXR). Liver X receptors (LXRs) respond to oxysterols derived from cholesterol and heterodimerize with RXRs to induce the transcription of ABCA1/G1 and inducible degrader of the LDLR (IDOL) through LXR response elements (LXREs). Estrogen binds to estrogen receptors (ERs) with unsubstantiated regulatory effects on lipid metabolism in T-cells. Crosstalk between ER and LXR has been reported in other cell types and transcription factor target site overlap has been reported for ERs with PPARs [PPRE/(ERE)] as well as with LXRs [LXRE/(ERE)]. TCR signaling: when T-cell receptors (TCRs) become antigen stimulated they associate with lipid rafts, plasma membrane microdomains enriched in glycosphingolipids (GSLs) and cholesterol. These lipid platforms enhance TCR activity by allowing signaling molecules such as lymphocyte-specific protein tyrosine kinase (Lck) to associate with the TCR and phosphorylate activation motifs for downstream signaling. Altering membrane raft lipid composition modifies TCR signaling and therefore T-cell functions. Manipulating nuclear receptors may control T-cell function in autoimmunity and cancer. This image was produced using images from Servier Medical Art, licensed under a Creative Common Attribution 3.0 Generic License http://smart.servier.com.

Cholesterol has a fundamental role in almost every aspect of mammalian physiology and consequently its levels are tightly regulated by multiple mechanisms modulating its endogenous synthesis, uptake, storage, efflux to the circulation and trafficking through intracellular compartments (31). When these fail, cholesterol metabolism becomes dysregulated resulting in toxicity both at a cellular and systemic level. As described below, sterol metabolism is not only important to determine metabolic homeostasis but is also a crucial regulator of immune cell function (32). The transcription factors LXRα and LXRβ lie at the interface between cholesterol metabolism and immune function (33). LXRs are primarily expressed in metabolically active cells and tissues such as the liver and intestine as well as in macrophages. Both LXRα and LXRβ are endogenously activated by certain oxysterols or oxidized forms of cholesterol and are key to maintaining cellular cholesterol levels. LXRs do this through regulating the expression of metabolic mediators such as sterol transporters ATP-binding cassette transporters (ABCA1/ABCG1) (34) promoting reverse cholesterol transport and upregulation of the inducible degrader of the low density lipoprotein (LDL) receptor (IDOL), thereby suppressing LDL-mediated uptake (35, 36). LXRα/β both heterodimerize with retinoid X receptors (RXRs) to enable DNA binding and transcriptional regulation (Figure 1). The LXR/RXR heterodimer complex is permissive whereby either RXR or LXR ligands can enhance its transcriptional activity; LXRα deficiency in mice leads to systemic and cellular cholesterol overload and the development of metabolic conditions including atherosclerosis and steatosis (33). LXRs also regulate fatty acid synthesis through the induction of sterol regulatory element-binding protein 1c (SREBP1c) and fatty acid synthase (FASYN) (33).

Liver X receptors also modulate a broad spectrum of immune responses (37). In murine macrophages, LXR stimulation alters membrane phospholipid composition by inducing the expression of lysophosphatidylcholine acyltransferase 3 (LPCAT3) which incorporates free polyunsaturated fatty acids into phospholipids (38) and reduces membrane cholesterol content by promoting cholesterol efflux *via* ABCA1, leading to changes in membrane order/fluidity and the attenuation of inflammatory pathways (39). These LXR-mediated changes in macrophage PM lipid composition and fluidity disrupt toll-like receptor (TLR) signaling pathways and inhibit downstream nuclear factor kappa B (NF-κB) and mitogen-activated protein kinase (MAPK) proinflammatory signaling thus dampening inflammation.

To date, most studies investigating the role of LXRs in modulating immunity *via* altering PM lipid composition have been conducted in murine cells and macrophages and, it remains to be examined whether these mechanisms are similarly regulated in human T-cells (40).

### Estrogen Receptors (ERs)

Males and females differ in their immune response to foreign and self-antigens and consequently they differ in their risk of infection and prevalence of autoimmune diseases; males are generally more susceptible to infections than females and females represent ~80% of all patients with autoimmunity (41). The mechanisms underlying this sexual dimorphism remain largely unresolved (42). It is known that fundamental differences exist in the frequency and activity of T-cell subsets by gender across multiple ethnicities (43–45). Notably, some gender differences in adaptive immune responses are present throughout life, while others are manifested following the onset of puberty and prior to reproductive senescence implicating both genetic and hormonal influences (42). However, little is known about the regulation of lipid metabolism by estrogen (E2), particularly in immune cells. A recent study in mice showed the reproductive cycle determines the size and efficiency of hepatic high-density lipoprotein (HDL) particles with regards to their cholesterol efflux capacity. More efficient atheroprotective HDL is produced during high E2 phases of the menstrual cycle, resulting in increased cholesterol efflux capacity (46). This may alter the levels of cholesterol in the PM and consequently the composition of PM lipid rafts, as has been shown in antigen-presenting cells (APCs) (47), thereby influencing proinflammatory signaling. This effect on lipid metabolism is mediated by estrogen receptor-α (ERα) control of LXRα transcriptional activity through the binding of the receptors to promoters or enhancer regions of LXRα target genes involved in cholesterol homeostasis. These genes included *Abca1* and *Abcg5*. E2-bound ERα was suggested to promote LXR binding to these genes thereby inducing their transcriptional activation (46) (Figure 1). In addition, it was shown that LXRα stimulation in transgenic mice resulted in increased urinary secretion of biliary acids in females only, again suggesting crosstalk between LXR and ER activation (48). Interestingly, regulatory crosstalk between LXRβ and ERα within lipid rafts affecting intracellular signaling to promote nitric oxide production was previously reported in endothelial cells (49). It is currently unknown whether E2 also regulates lipid metabolism in immune cells. Interestingly, it has been shown that in cancer cells hydroxylated derivatives of cholesterol such as 25-hydroxycholesterol can selectively modulate ER activity (50) and rescue the antiproliferative effects of fulvestrant, an ER antagonist (51). This again demonstrates a cross-talk between lipid metabolism and hormone receptors exists in other cellular systems (51).

The differential effects of E2 on immune function (42) reflect not only variation in hormone concentrations but also the expression, localization and ER subtype composition in immune cells. These nuclear receptors can also be found palmitoylated at the PM and modulate E2-induced non-genomic signaling (MAPK/extracellular signal-regulated kinase pathway) (52) (Figure 1). The two classical ERs (ERα and ERβ) dimerize in response to estrogen, and bind to estrogen response elements (EREs) in transcriptional regulatory regions in their target genes. A study utilizing specific ERα functional knockouts identified tissue-specific roles for the nuclear and membrane ERα forms. It appears the membrane bound form was important for ovarian function and the nuclear form for uterine responses to estrogen (53). Therefore, ER location may be important in controlling T-cell metabolism and function. Another form of ER has been described, the G-protein-coupled estrogen receptor (GPER30), which is exclusively PM bound and associated with lipid rafts (54). GPER30 induces non-genomic intracellular signaling independent of ERα and ERβ and can influence cell proliferation, survival, differentiation and metabolism (55, 56). ERα36 is a splice variant of ERα lacking transcriptional activation domains that resides at the PM but is also found in the cytoplasm and nucleus (57), where it can inhibit NF-κB, thereby reducing interleukin (IL)-6 expression (58). The role of the different ERs in human immunity remains unresolved. Genetic deficiency of ERα in murine models of systemic lupus erythematosus (SLE) significantly decreases disease severity and prolongs survival, while ERβ deficiency has minimal to no effect in animal models of autoimmunity (59).

#### Sterol Regulatory Element-Binding Proteins

Sterol regulatory element-binding proteins are another family of transcription factors that sense cholesterol levels and consequently reprogram lipid metabolism. SREBPs reside in the endoplasmic reticulum, until they are activated by low cholesterol levels, which trigger their transport to the Golgi complex where they are proteolytically modified to their active nuclear form (60). In the nucleus, they promote the transcription of genes associated with production of cellular cholesterol or fatty acid levels. There are two mammalian genes for SREBP, *SREBF1* and *SREBF2*. *SREBF1* is transcribed as two isoforms, SREBP1a and SREBP1c, both of which are involved in synthesis (through FASYN) and metabolism of fatty acids (Figure 1) (61). SREBP2 regulates cellular cholesterol levels by enhancing the transcription of its target genes including hydroxymethylglutaryl (HMG)-CoA reductase (*HMGCoR*) and the LDL receptor (*LDLR*); involved in cellular cholesterol synthesis and uptake, respectively. There is however a vast overlap between the function of the SREBPs (60–62). Cholesterol and its hydroxylated derivatives inhibit the transport of SREBPs to the Golgi complex (63). Interestingly, endogenous oxysterol ligands for LXR have the dual effect of inhibiting the processing of SREBP to its active form in addition to inducing SREBP transcription, demonstrating a potent feedback loop for the regulation of intracellular cholesterol levels (64). In cancer cells it has been shown that FASYN drives the synthesis of phospholipids that become integrated into membrane lipid rafts resulting in altered regulation of membrane composition and loss of cell function (65).

#### Peroxisome Proliferator-Activated Receptors (PPARs)

Peroxisome proliferator-activated receptors are also key players in the transcriptional regulation of lipid metabolism. The three subtypes PPARα, PPARγ, and PPARδ have a variety of roles in response to activation by their ligands, which include fatty acids (66). PPARα is primarily expressed in tissues that carry out large amounts of fatty acid oxidation such as the kidney and liver. PPARα upregulates apolipoprotein A-I and apolipoprotein A-II (APOAI/II) resulting in an increase in circulating HDL cholesterol and enhances the expression of genes associated with triglyceride metabolism. Similarly, PPARγ modulates fatty acid transport and uptake *via* fatty acid transport proteins (FATP) and CD36, respectively, but is more commonly expressed in adipose tissue where it plays a crucial role in adipogenesis (67). PPARδ is less well studied, but is found in multiple metabolic tissues including adipose, liver and skeletal muscle (68) where it plays a role in β-oxidation of fatty acids, cholesterol efflux and glucose homeostasis (68). As with LXRs, PPARs require heterodimerization with RXRs to function as transcription factors; these complexes recognize PPAR response elements (PPREs) (66) in regulatory sequences present in their target genes (Figure 1). Oral administration of these agonists reduced clinical symptoms in an experimental model of autoimmunity (69). Despite the documented roles of PPARs in cholesterol and fatty acid metabolism in metabolic tissues, very little is known about PPAR-regulation of these pathways in immune cells. In the macrophage THP-1 cell line, PPARγ induces cellular cholesterol *via* the direct upregulation of HMG-CoA reductase cholesterol synthesis enzyme (70). Additionally, PPARδ stimulation increases PM cholesterol levels in malignant B-cells, although an equivalent role in T-cells remains to be established (71). In contrast, PPARδ agonists in macrophages increase reverse cholesterol transport *via* the upregulation of ABCA1, thus lowering cellular cholesterol levels (72). To date, the effect of PPAR activation on lipid raft composition has not been studied, but it is intriguing to speculate that changes in fatty acid levels and their availability could influence *de novo* GSL synthesis. Likewise, changes in cholesterol biosynthesis and/or efflux could affect intracellular and membrane cholesterol levels and thus the fluidity of the cell membrane.

Interestingly, a potential cross-talk between PPARα and ERs has been suggested. Elevated expression of ERα and ERβ reduced PPRE-mediated gene transcription, suggesting that ERs may bind to the PPREs in those regulated genes (73). Notably, there is also evidence suggesting that PPAR/RXR heterodimers can bind directly to EREs (73–75). The response elements of these nuclear receptors contain a similar half-site which could allow binding of either receptor (74). The inhibitory effects of increased ERs on PPAR-mediated gene regulation could also be due to increased competition for transcriptional coactivators (76–78). Additionally, a study has reported sexually dimorphic genome-wide binding of RXRα in mice and identified 44 male- and 43 female-dominant RXR target genes in liver. Importantly, many of those genes predominantly regulated in females were involved in fatty acid metabolism including *Faysn* and stearoyl-CoA desaturase 1 (*Scd1)*, suggesting a role for RXR function in modulating gender-specific lipid metabolism (79). This may influence many aspects of metabolism through RXR heterodimerization with LXRs and/or PPARs. Together, these studies suggest that the modulation of membrane lipids by these transcription factors may be sexually dimorphic which will need to be considered by future studies.

## Lipid Metabolism Regulators and T-Cell PM Lipid Raft Composition

The tightly controlled network of transcriptionally regulated lipids described above could be critical for T-cell function *via* maintaining lipid raft homeostasis and influencing T-cell signaling pathways as summarized in Table 1 (2).

**Table 1:** Current studies linking T-cell function with nuclear receptor modulation of lipid metabolism.

Nuclear receptor	Lipids	Influence on T-cell function	Disease implication	Reference
LXRβ,	GSL, cholesterol	Altered TCR signaling, reduced proliferation, inhibition of Th1 and Th17 and induction of Treg differentiation	Atherosclerosis, multiple sclerosis, arthritis, type 1 diabetes, SLE	(15, 80–82)
SREBPs	Fatty acids, cholesterol	CD8^+^ T-cell clonal expansion, CD8^+^ cytotoxicity	Hyperlipidemia, diabetes, atherosclerosis	(80, 84, 110)
PPARα	Fatty acids, cholesterol	IL-4 secretion, IFNγ, proliferation	Atherosclerosis, hypertriglyceridemia, hypoalphalipoproteinemia, diabetes, autoimmune encephalomyelitis	(85)
PPARγ	Fatty acids, cholesterol	Proliferation, IL-2 secretion, apoptosis	Atherosclerosis, hypertriglyceridemia, hypoalphalipoproteinemia, diabetes, autoimmune myocarditis	(87, 88)
PPARδ	Fatty acids, cholesterol	Proliferation, reduced proapoptotic effect of type 1 interferons, IFN-γ, and IL-17 secretion	Atherosclerosis, hypertriglyceridemia, hypoalphalipoproteinemia, diabetes, SLE	(86)
ERα	Cholesterol, fatty acids	All PPAR and LXR effects through cross-talk		(46, 73, 74)

LXRβ is the predominantly active form of LXR in T-cells (80). LXRβ influences T-cell proliferation through ABCG1-dependent regulation of intracellular cholesterol thereby affecting antigen-specific immune responses (80). It is likely that this effect is driven by reducing PM cholesterol that disrupts lipid raft-associated TCR signaling. In addition, our work identified that lipid raft-associated GSLs correlate with enhanced levels of LXRβ and LXR-modulated cholesterol trafficking proteins Niemann-Pick type C 1 and 2 (NPC1/2) in human CD4^+^ T-cells from autoimmune disease patients (15), although it remains to be elucidated whether LXR directly regulates GSLs in T-cell subsets from healthy individuals. LXR stimulation *in vitro* inhibits Th1 and Th17 cytokine production and induces regulatory T-cell polarization suggesting a role for LXR-driven lipid modulation in anti-inflammatory T-cell differentiation potentially by reducing PM cholesterol *via* increased cholesterol efflux (81, 82).

The mechanism of action of SREBPs is also particularly important in T-cell function as cholesterol homeostasis is critical to PM lipid raft composition and fatty acids provide an abundant T-cell energy source (83). For instance, CD8^+^ T-cells are unable to undergo clonal expansion in response to viral infection when SREBPs are not present, which can be rescued by supplementation with cholesterol (84).

All three PPAR subsets are expressed in T-cells where they are involved in both metabolic regulation and inflammation (66, 85, 86). PPAR modulation of cholesterol may play a role in regulating lipid rafts and therefore TCR signaling and their role in fatty acid oxidation likely alters T-cell energy sources. PPAR-mediated upregulation of ApoAI/II in the periphery may indirectly influence T-cell cholesterol levels *via* elevated HDL levels and increased cholesterol efflux. In addition, these factors have been shown to affect cell death and proliferation. Activation of PPARγ in helper T-cells suppresses proliferation, IL-2 expression and induce apoptosis (87, 88). PPARα antagonizes NF-κB in T-cells, and conversely T-cell activation results in reduced PPARα expression (85). PPARα agonists increase IL-4 secretion, inhibit interferon (IFN)-γ expression, and reduce the proliferation of human T-cell lines. Stimulation of PPARδ increases T-cell proliferation and reduces the proapoptotic effect of type 1 IFNs (86). In an experimental autoimmune disease model, PPARδ stimulation reduced IFN-γ and IL-17 secretion from T-cells (89). This suggests possible PPAR regulatory actions on T-cell differentiation through modification of lipid metabolism.

Due to the striking gender bias in autoimmunity (90) and reported differences in T-cell function, it is important to consider gender in this area of research. The two classical ERs (ERα and ERβ) exhibit differential expression; ERα is more highly expressed in T-cells than ERβ (91). Altered ER profiles could contribute to differences in PM-associated E2 signaling in T-cell subsets and between genders. Cross-talk between ERs and LXRs may also play a role in the lipid modification of T-cells and therefore function. Interestingly, there is evidence to suggest that gender and/or estrogen are able to modulate PPAR function. Dunn et al. demonstrated that male mice express more PPARα than females and that this differential expression is hormone sensitive. Furthermore genetic ablation of the PPARα gene resulted in the loss of antagonism of NF-κB, increased production of Th1 and decreased production of Th2 cytokines by T-cells. This genetic ablation in an experimental model of autoimmune encephalomyelitis increased clinical symptoms in male but not female mice (92). This suggests a sex-specific sensitivity to the protective actions of PPARα relevant to the gender bias seen in autoimmunity.

## Therapeutic Targeting

The tight network of transcriptional metabolic regulators described above provides a great opportunity for therapeutic targeting (Table 1). Because of the cross-talk between these different nuclear receptors and pathways, manipulating multiple receptors could represent an effective strategy. The SREBP pathway responds to low cholesterol, and therefore the use of statins, which inhibit the cholesterol synthesis enzyme HMG-CoA reductase, secondarily increases the activity of SREBPs in an attempt to increase cellular cholesterol and fatty acid levels. From an autoimmune perspective, statins could be used therapeutically to counter the pathogenic increase in T-cell lipid rafts through lowering membrane cholesterol. *In vitro* culture of T-cells with atorvastatin reduces T-cell signaling from lipid rafts, ultimately reducing IL-6 production implicated in SLE pathogenesis (29). It has been shown that statins alter the ratio of pro- and anti-inflammatory responder T-cells, inhibit Th1 differentiation and reduce the activation and migration of CD4^+^ autoreactive T-cells across the blood–brain barrier in multiple sclerosis (93–95). This finding supports an important role for cholesterol metabolism in T-cell function. Notably, simvastatin has shown promise in a phase 2 trial in people with multiple sclerosis; the drug reduced the annual rate of whole-brain atrophy without adverse side effects (96). Independent of their modulation of cholesterol, statins may also influence T-cell function through the inhibition of prenylation (geranylgeranylation or farnesylation) (97). Prenylation of GTPases of the Ras and Rac subfamilies allows their targeting to the cell membrane which is integral to TCR signaling (98, 99). Alternatively, inhibiting SREBPs may counteract overactive TCR signaling. A small molecule SREBP processing inhibitor named betulin has been shown to improve hyperlipidemia and insulin resistance and reduces atherosclerotic plaques (100). SREBP inhibition also prevents CD8^+^ T-cell expansion in response to viral infection (84). Another potential therapeutic target is the LXRs. Synthetic ligands that stimulate the activity of these receptors exist which reduce cellular and membrane cholesterol content. An example of this is the non-steroidal ligand GW3965, an LXR agonist that has been shown to modulate macrophage, dendritic cell and T-cell function (51, 80, 101). However, the value of these therapeutics has not been explored extensively in T-cells. In light of the evidence that activated ERs aid the transcriptional function of LXRs, interact with LXRs in lipid rafts in endothelial cells, and respond to oxysterols, it is plausible to hypothesize that LXR therapeutics could be more effective in premenopausal women although this is something that has not been explored to date. Synthetic LXR ligands have been investigated as anti-atherosclerotic agents in experimental models of atherosclerosis and in a human phase 1 trial (102, 103). The main obstacle encountered in the development of LXR ligands as clinical therapeutic agents in human metabolic diseases is the concomitant increase in liver triglycerides by these agents, an effect primarily mediated by LXRα (104, 105). Furthermore, LXR activation is gaining interest in the fight against cancer because of their actions on cholesterol metabolism in cancer cells coupled with their effects on cell proliferation, growth arrest and apoptosis (106). Some of these aspects have been described for CD8^+^ T-cells (80). Whether this is recapitulated in other immune cell subsets and the impact of this in female-predominant autoimmune diseases needs to be established. Altogether this emphasizes the need for a greater understanding of isoform- (LXRα vs. LXRβ) and tissue/cell type-specific effects of LXRs in health and disease.

Peroxisome proliferator-activated receptor pharmaceutical agonists including fibrates for PPARα, glitazones for PPARγ, and phenoxyacetic acid derivatives for PPARδ have therapeutic value in hypertriglyceridemia, hypoalphalipoproteinemia, and diabetes (67, 68, 107). PPARα activators reduce Th1 and increase Th2 polarization making these therapeutics attractive for the treatment of autoimmune diseases (69). PPARγ agonists have also shown promise following a study of autoimmune myocarditis in Lewis rats. A PPARγ agonist ameliorated disease severity, which was also attributed to a Th1/2 phenotypic switch (108). It will be interesting to assess the effect of PPARs on membrane lipids, especially as Th1/Th2 status has been linked to differences in PM order (9). Again, gender may play a role in the effectiveness of these treatments. Activated ERs may compete for PPAR DNA binding and there is evidence to suggest that PPAR ligands perform better under estrogen free/ER-inhibited conditions (77). Therefore, inverse to the LXR hypothesis, PPAR therapies may be of greater benefit in males and post-menopausal women. Finally, in recent years, modulation of PM lipid composition and structure, either by reducing or by increasing PM cholesterol levels, has been investigated in the treatment of cancer. Reduced PM cholesterol has been associated with increased cancer cell metastasis whereas high PM cholesterol has been linked to drug resistance. In these contexts, lipid modulating therapies combined with conventional drugs can improve the efficacy of anti-cancer treatments (109). Recently, Avasimibe, a drug that blocks free cholesterol esterification and its subsequent storage as cellular lipid droplets by inhibiting the enzyme acetyl-CoA acetyltransferase 1, increased the efficacy of checkpoint inhibitor blockade in preclinical models of melanoma and lung carcinoma. This was achieved by increased PM cholesterol leading to stronger TCR signaling and cytotoxic activity in CD8^+^ T-cells (110, 111). This supports the possibility of combining established therapeutics with lipid-modulating treatments in order to enhance efficacy and improve outcomes in a range of clinical settings.

## Conclusion and Perspectives

Here, we have summarized evidence showing that manipulation of lipid metabolism in T-cells by targeting nuclear receptor transcription factors could be a promising therapeutic avenue in the treatment of autoimmune diseases. However, the cross-talk between this tight network of receptors and transcription factors will need to be considered when determining which receptors to target. We have also highlighted that gender is an important factor for consideration, thus emphasizing the relevance of these receptors in a group of immune diseases dominated by gender bias. With the advent of advanced lipidomic technologies, we anticipate that in the coming years more in depth studies on PM lipid composition and its metabolic, inflammatory and pharmacological regulation in different immune cell types including T-cells will become available. This will likely allow new opportunities to use ligands targeting these receptors/factors as adjuvant therapies in various proliferative and immunological disorders.

## Author Contributions

All authors listed have made a substantial, direct, and intellectual contribution to the work and approved it for publication.

## Conflict of Interest Statement

The authors declare that they have no commercial or financial relationships that could be construed as a potential conflict of interest relating to this work.

## Abbreviations

PM, plasma membrane; LXR, liver-X-receptor; PPAR, peroxisome proliferator-activated receptor; ER, estrogen receptor; SREBP, sterol regulatory element-binding proteins; ABCA1/G1, ATP-binding cassette transporter A1/G1; HDL, high-density lipoprotein; LDL, low-density lipoprotein; LDLR, low-density lipoprotein receptor; NF-κB, nuclear factor kappa B; FAS, fatty acid synthesis; FAYSN, fatty acid synthase; FAO, fatty acid oxidation; HMG-CoAR, hydroxymethylglutaryl-coenzyme-A reductase; RXR, retinoid-X-receptor; TCR, T-cell receptor; TLR, toll-like receptor; GSL, glycosphingolipid; LPCAT3, lysophosphatidylcholine acyltransferase 3; MAPK, mitogen-activated protein kinase; APOA/G1, apolipoprotein A1/G1; IFN, interferon; IL, interleukin; PPRE, PPAR response element; ERE, estrogen response element; IDOL, inducible degrader of the low-density lipoprotein receptor; SRE, sterol response element; LXRE, LXR response element.

## References

[1] Dimeloe S Burgener AV Grählert J Hess C. T-cell metabolism governing activation, proliferation and differentiation; a modular view. Immunology (2017) 150(1):35–44. 10.1111/imm.12655 27479920 PMC5341500

[2] Waddington KE Jury EC. Manipulating membrane lipid profiles to restore T-cell function in autoimmunity. Biochem Soc Trans (2015) 43(4):745–51. 10.1042/BST20150111 26551723

[3] Wu W Shi X Xu C. Regulation of T cell signalling by membrane lipids. Nat Rev Immunol (2016) 16(11):690–701. 10.1038/nri.2016.103 27721483

[4] Simons K. Cell membranes: a subjective perspective. Biochim Biophys Acta (2016) 1858(10):2569–72. 10.1016/j.bbamem.2016.01.023 26827711

[5] Lingwood D Simons K Lipid rafts as a membrane-organizing principle. Science (2010) 327(5961):46–50. 10.1126/science.1174621 20044567

[6] Jury EC Flores-Borja F Kabouridis PS. Lipid rafts in T cell signalling and disease. Semin Cell Dev Biol (2007) 18(5):608–15. 10.1016/j.semcdb.2007.08.002 17890113 PMC2596300

[7] Megha Bakht O London E. Cholesterol precursors stabilize ordinary and ceramide-rich ordered lipid domains (lipid rafts) to different degrees. Implications for the Bloch hypothesis and sterol biosynthesis disorders. J Biol Chem (2006) 281(31):21903–13. 10.1074/jbc.M600395200 16735517

[8] Janes PW Ley SC Magee AI Kabouridis PS. The role of lipid rafts in T cell antigen receptor (TCR) signalling. Semin Immunol (2000) 12(1):23–34. 10.1006/smim.2000.0204 10723795

[9] Miguel L Owen DM Lim C Liebig C Evans J Magee AI Primary human CD4(+) T cells have diverse levels of membrane lipid order that correlate with their function. J Immunol (2011) 186(6):3505–16. 10.4049/jimmunol.1002980 21307290

[10] Araldi E Fernández-Fuertes M Canfrán-Duque A Tang W Cline GW Madrigal-Matute J Lanosterol modulates TLR4-mediated innate immune responses in macrophages. Cell Rep (2017) 19(13):2743–55. 10.1016/j.celrep.2017.05.093 28658622 PMC5553565

[11] Levental KR Lorent JH Lin X Skinkle AD Surma MA Stockenbojer EA Polyunsaturated lipids regulate membrane domain stability by tuning membrane order. Biophys J (2016) 110(8):1800–10. 10.1016/j.bpj.2016.03.012 27119640 PMC4850323

[12] Swamy M Beck-Garcia K Beck-Garcia E Hartl FA Morath A Yousefi OS A cholesterol-based allostery model of T cell receptor phosphorylation. Immunity (2016) 44(5):1091–101. 10.1016/j.immuni.2016.04.011 27192576

[13] Molnár E Swamy M Holzer M Beck-García K Worch R Thiele C Cholesterol and sphingomyelin drive ligand-independent T-cell antigen receptor nanoclustering. J Biol Chem (2012) 287(51):42664–74. 10.1074/jbc.M112.386045 23091059 PMC3522267

[14] Wang F Beck-García K Zorzin C Schamel WW Davis MM. Inhibition of T cell receptor signaling by cholesterol sulfate, a naturally occurring derivative of membrane cholesterol. Nat Immunol (2016) 17(7):844–50. 10.1038/ni.3462 27213689 PMC4916016

[15] McDonald G Deepak S Miguel L Hall CJ Isenberg DA Magee AI Normalizing glycosphingolipids restores function in CD4+ T cells from lupus patients. J Clin Invest (2014) 124(2):712–24. 10.1172/JCI69571 24463447 PMC3904606

[16] Cho JH Kim HO Surh CD Sprent J. T cell receptor-dependent regulation of lipid rafts controls naive CD8+ T cell homeostasis. Immunity (2010) 32(2):214–26. 10.1016/j.immuni.2009.11.014 20137986 PMC2830358

[17] Zhu Y Gumlaw N Karman J Zhao H Zhang J Jiang JL Lowering glycosphingolipid levels in CD4+ T cells attenuates T cell receptor signaling, cytokine production, and differentiation to the Th17 lineage. J Biol Chem (2011) 286(17):14787–94. 10.1074/jbc.M111.218610 21402703 PMC3083190

[18] Nagafuku M Okuyama K Onimaru Y Suzuki A Odagiri Y Yamashita T CD4 and CD8 T cells require different membrane gangliosides for activation. Proc Natl Acad Sci U S A (2012) 109(6):E336–42. 10.1073/pnas.1114965109 22308377 PMC3277553

[19] Degroote S Wolthoorn J van Meer G. The cell biology of glycosphingolipids. Semin Cell Dev Biol (2004) 15(4):375–87. 10.1016/j.semcdb.2004.03.007 15207828

[20] Levental I Grzybek M Simons K. Raft domains of variable properties and compositions in plasma membrane vesicles. Proc Natl Acad Sci U S A (2011) 108(28):11411–6. 10.1073/pnas.1105996108 21709267 PMC3136254

[21] Nazarov-Stoica C Surls J Bona C Casares S Brumeanu TD. CD28 signaling in T regulatory precursors requires p56lck and rafts integrity to stabilize the Foxp3 message. J Immunol (2009) 182(1):102–10. 10.4049/jimmunol.182.1.102 19109140

[22] Balamuth F Leitenberg D Unternaehrer J Mellman I Bottomly K. Distinct patterns of membrane microdomain partitioning in Th1 and Th2 cells. Immunity (2001) 15(5):729–38. 10.1016/S1074-7613(01)00223-0 11728335

[23] Köberlin MS Snijder B Heinz LX Baumann CL Fauster A Vladimer GI A conserved circular network of coregulated lipids modulates innate immune responses. Cell (2015) 162(1):170–83. 10.1016/j.cell.2015.05.051 26095250 PMC4523684

[24] Griffié J Shannon M Bromley CL Boelen L Burn GL Williamson DJ A Bayesian cluster analysis method for single-molecule localization microscopy data. Nat Protoc (2016) 11(12):2499–514. 10.1038/nprot.2016.149 27854362

[25] Rubin-Delanchy P Burn GL Griffié J Williamson DJ Heard NA Cope AP Bayesian cluster identification in single-molecule localization microscopy data. Nat Methods (2015) 12(11):1072–6. 10.1038/nmeth.3612 26436479

[26] Owen DM Rentero C Magenau A Abu-Siniyeh A Gaus K Quantitative imaging of membrane lipid order in cells and organisms. Nat Protoc (2012) 7(1):24–35. 10.1038/nprot.2011.419 22157973

[27] Ashdown GW Owen DM Imaging membrane order using environmentally sensitive fluorophores. 2nd ed In: Owen DM, editor. Methods in Membrane Lipids. Totowa: Humana Press Inc (2015). p. 115–22. 10.1007/978-1-4939-1752-5_10 25331132

[28] Owen DM Gaus K. Imaging lipid domains in cell membranes: the advent of super-resolution fluorescence microscopy. Front Plant Sci (2013) 4:9. 10.3389/fpls.2013.00503 24376453 PMC3859905

[29] Jury EC Isenberg DA Mauri C Ehrenstein MR. Atorvastatin restores Lck expression and lipid raft-associated signaling in T cells from patients with systemic lupus erythematosus. J Immunol (2006) 177(10):7416–22. 10.4049/jimmunol.177.10.7416 17082661

[30] Jury EC Kabouridis PS Flores-Borja F Mageed RA Isenberg DA. Altered lipid raft-associated signaling and ganglioside expression in T lymphocytes from patients with systemic lupus erythematosus. J Clin Invest (2004) 113(8):1176–87. 10.1172/JCI200420345 15085197 PMC385405

[31] Mesmin B Maxfield FR. Intracellular sterol dynamics. Biochim Biophys Acta (2009) 1791(7):636–45. 10.1016/j.bbalip.2009.03.002 19286471 PMC2696574

[32] Spann NJ Glass CK. Sterols and oxysterols in immune cell function. Nat Immunol (2013) 14(9):893–900. 10.1038/ni.2681 23959186

[33] Kidani Y Bensinger SJ. Liver X receptor and peroxisome proliferator-activated receptor as integrators of lipid homeostasis and immunity. Immunol Rev (2012) 249(1):72–83. 10.1111/j.1600-065X.2012.01153.x 22889216 PMC4007066

[34] Repa JJ Turley SD Lobaccaro JA Medina J Li L Lustig K Regulation of absorption and ABC1-mediated efflux of cholesterol by RXR heterodimers. Science (2000) 289(5484):1524–9. 10.1126/science.289.5484.1524 10968783

[35] Fievet C Staels B Liver X receptor modulators: effects on lipid metabolism and potential use in the treatment of atherosclerosis. Biochem Pharmacol (2009) 77(8):1316–27. 10.1016/j.bcp.2008.11.026 19101522

[36] Zelcer N Hong C Boyadjian R Tontonoz P. LXR regulates cholesterol uptake through idol-dependent ubiquitination of the LDL receptor. Science (2009) 325(5936):100–4. 10.1126/science.1168974 19520913 PMC2777523

[37] Steffensen KR Jakobsson T Gustafsson J-Å. Targeting liver X receptors in inflammation. Expert Opin Ther Targets (2013) 17(8):977–90. 10.1517/14728222.2013.806490 23738533

[38] Rong X Albert CJ Hong C Duerr MA Chamberlain BT Tarling EJ LXRs regulate ER stress and inflammation through dynamic modulation of membrane phospholipid composition. Cell Metab (2013) 18(5):685–97. 10.1016/j.cmet.2013.10.002 24206663 PMC3889491

[39] Ito A Hong C Rong X Zhu X Tarling EJ Hedde PN LXRs link metabolism to inflammation through Abca1-dependent regulation of membrane composition and TLR signaling. eLife (2015) 4:e08009 10.7554/eLife.08009 26173179 PMC4517437

[40] Waddington KE Jury EC Pineda-Torra I. Liver X receptors in immune cell function in humans. Biochem Soc Trans (2015) 43(4):752–7. 10.1042/BST20150112 26551724

[41] Klein SL Flanagan KL Sex differences in immune responses. Nat Rev Immunol (2016) 16(10):626–38. 10.1038/nri.2016.90 27546235

[42] Hughes GC Choubey D. Modulation of autoimmune rheumatic diseases by oestrogen and progesterone. Nat Rev Rheumatol (2014) 10(12):740–51. 10.1038/nrrheum.2014.144 25155581

[43] Uppal SS Verma S Dhot PS Normal values of CD4 and CD8 lymphocyte subsets in healthy Indian adults and the effects of sex, age, ethnicity, and smoking. Cytometry B Clin Cytom (2003) 52B(1):32–6. 10.1002/cyto.b.10011 12599179

[44] Sankaran-Walters S Macal M Grishina I Nagy L Goulart L Coolidge K Sex differences matter in the gut: effect on mucosal immune activation and inflammation. Biol Sex Differ (2013) 4:12. 10.1186/2042-6410-4-10 23651648 PMC3652739

[45] Lisse IM Aaby P Whittle H Jensen H Engelmann M Christensen LB. T-lymphocyte subsets in West African children: impact of age, sex, and season. J Pediatr (1997) 130(1):77–85. 10.1016/S0022-3476(97)70313-5 9003854

[46] Della Torre S Mitro N Fontana R Gomaraschi M Favari E Recordati C An essential role for liver ERalpha in coupling hepatic metabolism to the reproductive cycle. Cell Rep (2016) 15(2):360–71. 10.1016/j.celrep.2016.03.019 27050513 PMC4835581

[47] Wang SH Yuan SG Peng DQ Zhao SP. HDL and ApoA-I inhibit antigen presentation-mediated T cell activation by disrupting lipid rafts in antigen presenting cells. Atherosclerosis (2012) 225(1):105–14. 10.1016/j.atherosclerosis.2012.07.029 22862966

[48] Uppal H Saini SP Moschetta A Mu Y Zhou J Gong H Activation of LXRs prevents bile acid toxicity and cholestasis in female mice. Hepatology (2007) 45(2):422–32. 10.1002/hep.21494 17256725

[49] Ishikawa T Yuhanna IS Umetani J Lee WR Korach KS Shaul PW LXRβ/estrogen receptor-α signaling in lipid rafts preserves endothelial integrity. J Clin Invest (2013) 123(8):3488–97. 10.1172/JCI66533 23867501 PMC3726156

[50] Lappano R Recchia AG De Francesco EM Angelone T Cerra MC Picard D The cholesterol metabolite 25-hydroxycholesterol activates estrogen receptor alpha-mediated signaling in cancer cells and in cardiomyocytes. PLoS One (2011) 6(1):14 10.1371/journal.pone.0016631 PMC3031608 21304949

[51] Simigdala N Gao Q Pancholi S Roberg-Larsen H Zvelebil M Ribas R Cholesterol biosynthesis pathway as a novel mechanism of resistance to estrogen deprivation in estrogen receptor-positive breast cancer. Breast Cancer Res (2016) 18:14 10.1186/s13058-016-0713-5 27246191 PMC4888666

[52] Levin ER. Plasma membrane estrogen receptors. Trends Endocrinol Metab (2009) 20(10):477–82. 10.1016/j.tem.2009.06.009 19783454 PMC3589572

[53] Adlanmerini M Solinhac R Abot A Fabre A Raymond-Letron I Guihot AL Mutation of the palmitoylation site of estrogen receptor α in vivo reveals tissue-specific roles for membrane versus nuclear actions. Acta Physiol (2014) 211:61–61. 10.1073/pnas.1322057111 PMC3896153 24371309

[54] Gaudet HM Cheng SB Christensen EM Filardo EJ. The G-protein coupled estrogen receptor, GPER: the inside and inside-out story. Mol Cell Endocrinol (2015) 418(Pt 3):207–19. 10.1016/j.mce.2015.07.016 26190834

[55] Wang C Lv X He C Hua G Tsai MY Davis JS. The G-protein-coupled estrogen receptor agonist G-1 suppresses proliferation of ovarian cancer cells by blocking tubulin polymerization. Cell Death Dis (2013) 4:e869. 10.1038/cddis.2013.397 24136233 PMC3920961

[56] Wang C Dehghani B Li Y Kaler LJ Proctor T Vandenbark AA Membrane estrogen receptor regulates experimental autoimmune encephalomyelitis through up-regulation of programmed death 1. J Immunol (2009) 182(5):3294–303. 10.4049/jimmunol.0803205 19234228 PMC2729563

[57] Chaudhri RA Schwartz N Elbaradie K Schwartz Z Boyan BD Role of ERalpha36 in membrane-associated signaling by estrogen. Steroids (2014) 81:74–80. 10.1016/j.steroids.2013.10.020 24252378

[58] Pelekanou V Kampa M Kiagiadaki F Deli A Theodoropoulos P Agrogiannis G Estrogen anti-inflammatory activity on human monocytes is mediated through cross-talk between estrogen receptor ERalpha36 and GPR30/GPER1. J Leukoc Biol (2016) 99(2):333–47. 10.1189/jlb.3A0914-430RR 26394816

[59] Svenson JL EuDaly J Ruiz P Korach KS Gilkeson GS Impact of estrogen receptor deficiency on disease expression in the NZM2410 lupus prone mouse. Clin Immunol (2008) 128(2):259–68. 10.1016/j.clim.2008.03.508 18514033 PMC4778964

[60] Brown MS Goldstein JL The SREBP pathway: regulation of cholesterol metabolism by proteolysis of a membrane-bound transcription factor. Cell (1997) 89(3):331–40. 10.1016/S0092-8674(00)80213-5 9150132

[61] Horton JD Goldstein JL Brown MS SREBPs: activators of the complete program of cholesterol and fatty acid synthesis in the liver. J Clin Invest (2002) 109(9):1125–31. 10.1172/JCI0215593 11994399 PMC150968

[62] Guo D Bell EH Mischel P Chakravarti A. Targeting SREBP-1-driven lipid metabolism to treat cancer. Curr Pharm Des (2014) 20(15):2619–26. 10.2174/13816128113199990486 23859617 PMC4148912

[63] Ikonen E. Cellular cholesterol trafficking and compartmentalization. Nat Rev Mol Cell Biol (2008) 9(2):125–38. 10.1038/nrm2336 18216769

[64] Radhakrishnan A Ikeda Y Kwon HJ Brown MS Goldstein JL Sterol-regulated transport of SREBPs from endoplasmic reticulum to Golgi: oxysterols block transport by binding to Insig. Proc Natl Acad Sci U S A (2007) 104(16):6511–8. 10.1073/pnas.0700899104 17428920 PMC1851665

[65] Swinnen JV Van Veldhoven PP Timmermans L De Schrijver E Brusselmans K Vanderhoydonc F Fatty acid synthase drives the synthesis of phospholipids partitioning into detergent-resistant membrane microdomains. Biochem Biophys Res Commun (2003) 302(4):898–903. 10.1016/S0006-291X(03)00265-1 12646257

[66] Varga T Czimmerer Z Nagy L. PPARs are a unique set of fatty acid regulated transcription factors controlling both lipid metabolism and inflammation. Biochim Biophys Acta (2011) 1812(8):1007–22. 10.1016/j.bbadis.2011.02.014 21382489 PMC3117990

[67] Gervois P Torra IP Fruchart JC Staels B. Regulation of lipid and lipoprotein metabolism by PPAR activators. Clin Chem Lab Med (2000) 38(1):3–11. 10.1515/CCLM.2000.002 10774955

[68] Luquet S Gaudel C Holst D Lopez-Soriano J Jehl-Pietri C Fredenrich A Roles of PPAR delta in lipid absorption and metabolism: a new target for the treatment of type 2 diabetes. Biochim Biophys Acta (2005) 1740(2):313–7. 10.1016/j.bbadis.2004.11.011 15949697

[69] Lovett-Racke AE Hussain RZ Northrop S Choy J Rocchini A Matthes L Peroxisome proliferator-activated receptor alpha agonists as therapy for autoimmune disease. J Immunol (2004) 172(9):5790–8. 10.4049/jimmunol.172.9.5790 15100326

[70] Iida KT Kawakami Y Suzuki H Sone H Shimano H Toyoshima H PPAR gamma ligands, troglitazone and pioglitazone, up-regulate expression of HMG-CoA synthase and HMG-CoA reductase gene in THP-1 macrophages. FEBS Lett (2002) 520(1–3):177–81. 10.1016/S0014-5793(02)02811-9 12044893

[71] Sun L Shi Y Wang G Wang X Zeng S Dunn SE PPAR-delta modulates membrane cholesterol and cytokine signaling in malignant B cells. Leukemia (2017). 10.1038/leu.2017.162 28555083

[72] Oliver WR Jr Shenk JL Snaith MR Russell CS Plunket KD Bodkin NL A selective peroxisome proliferator-activated receptor delta agonist promotes reverse cholesterol transport. Proc Natl Acad Sci U S A (2001) 98(9):5306–11. 10.1073/pnas.091021198 11309497 PMC33205

[73] Wang X Kilgore MW Signal cross-talk between estrogen receptor alpha and beta and the peroxisome proliferator-activated receptor gamina1 in MDA-MB-231 and MCF-7 breast cancer cells. Mol Cell Endocrinol (2002) 194(1–2):123–33. 10.1016/S0303-7207(02)00154-5 12242035

[74] Keller H Givel F Perroud M Wahli W Signaling cross-talk between peroxisome proliferator-activated receptor retinoid-X receptor and estrogen-receptor through estrogen response elements. Mol Endocrinol (1995) 9(7):794–804. 10.1210/mend.9.7.7476963 7476963

[75] Nuñez SB Medin JA Braissant O Kemp L Wahli W Ozato K Retinoid X receptor and peroxisome proliferator-activated receptor activate an estrogen responsive gene independent of the estrogen receptor. Mol Cell Endocrinol (1997) 127(1):27–40. 10.1016/S0303-7207(96)03980-9 9099898

[76] Tcherepanova I Puigserver P Norris JD Spiegelman BM McDonnell DP. Modulation of estrogen receptor-alpha transcriptional activity by the coactivator PGC-1. J Biol Chem (2000) 275(21):16302–8. 10.1074/jbc.M001364200 10748020

[77] Jeong S Yoon M Inhibition of the actions of peroxisome proliferator-activated receptor A on obesity by estrogen. Obesity (2007) 15(6):1430–40. 10.1038/oby.2007.171 17557980

[78] Foryst-Ludwig A Clemenz M Hohmann S Hartge M Sprang C Frost N Metabolic actions of estrogen receptor beta (ER beta) are mediated by a negative cross-talk with PPAR gamma. PLoS Genet (2008) 4(6):16. 10.1371/journal.pgen.1000108 18584035 PMC2432036

[79] Kosters A Sun D Wu H Tian F Felix JC Li W Sexually dimorphic genome-wide binding of retinoid X receptor alpha (RXR alpha) determines male-female differences in the expression of hepatic lipid processing genes in mice. PLoS One (2013) 8(8):14 10.1371/journal.pone.0071538 PMC3747242 23977068

[80] Bensinger SJ Bradley MN Joseph SB Zelcer N Janssen EM Hausner MA LXR signaling couples sterol metabolism to proliferation in the acquired immune response. Cell (2008) 134(1):97–111. 10.1016/j.cell.2008.04.052 18614014 PMC2626438

[81] Walcher D Kümmel A Kehrle B Bach H Grüb M Durst R LXR activation reduces proinflammatory cytokine expression in human CD4-positive lymphocytes. Arterioscler Thromb Vasc Biol (2006) 26(5):1022–8. 10.1161/01.ATV.0000210278.67076.8f 16484597

[82] Herold M Breuer J Hucke S Knolle P Schwab N Wiendl H Liver X receptor activation promotes differentiation of regulatory T cells. PLoS One (2017) 12(9):13. 10.1371/journal.pone.0184985 28926619 PMC5604992

[83] Fox CJ Hammerman PS Thompson CB. Fuel feeds function: energy metabolism and the T-cell response. Nat Rev Immunol (2005) 5(11):844–52. 10.1038/nri1710 16239903

[84] Kidani Y Elsaesser H Hock MB Vergnes L Williams KJ Argus JP Sterol regulatory element-binding proteins are essential for the metabolic programming of effector T cells and adaptive immunity. Nat Immunol (2013) 14(5):489. 10.1038/ni.2570 23563690 PMC3652626

[85] Jones DC Ding XH Daynes RA Nuclear receptor peroxisome proliferator-activated receptor a (PPAR alpha) is expressed in resting murine lymphocytes – the PPAR alpha in T and B lymphocytes is both transactivation and transrepression competent. J Biol Chem (2002) 277(9):6838–45. 10.1074/jbc.M106908200 11726654

[86] al Yacoub N Romanowska M Krauss S Schweiger S Foerster J. PPAR delta is a type 1 IFN target gene and inhibits apoptosis in T cells. J Invest Dermatol (2008) 128(8):1940–9. 10.1038/jid.2008.32 18305567

[87] Clark RB Bishop-Bailey D Estrada-Hernandez T Hla T Puddington L Padula SJ. The nuclear receptor PPAR gamma and immunoregulation: PPAR gamma mediates inhibition of helper T cell responses. J Immunol (2000) 164(3):1364–71. 10.4049/jimmunol.164.3.1364 10640751

[88] Harris SG Phipps RP. The nuclear receptor PPAR gamma is expressed by mouse T lymphocytes and PPAR gamma agonists induce apoptosis. Eur J Immunol (2001) 31(4):1098–105. 10.1002/1521-4141(200104)31:4<1098::AID-IMMU1098>3.0.CO;2-I 11298334

[89] Kanakasabai S Chearwae W Walline CC Iams W Adams SM Bright JJ. Peroxisome proliferator-activated receptor delta agonists inhibit T helper type 1 (Th1) and Th17 responses in experimental allergic encephalomyelitis. Immunology (2010) 130(4):572–88. 10.1111/j.1365-2567.2010.03261.x 20406305 PMC2913268

[90] Ngo ST Steyn FJ McCombe PA. Gender differences in autoimmune disease. Front Neuroendocrinol (2014) 35(3):347–69. 10.1016/j.yfrne.2014.04.004 24793874

[91] Phiel KL Henderson RA Adelman SJ Elloso MM. Differential estrogen receptor gene expression in human peripheral blood mononuclear cell populations. Immunol Lett (2005) 97(1):107–13. 10.1016/j.imlet.2004.10.007 15626482

[92] Dunn SE Ousman SS Sobel RA Zuniga L Baranzini SE Youssef S Peroxisome proliferator-activated receptor (PPAR)alpha expression in T cells mediates gender differences in development of T cell-mediated autoimmunity. J Exp Med (2007) 204(2):321–30. 10.1084/jem.20061839 17261635 PMC2118721

[93] Youssef S Stüve O Patarroyo JC Ruiz PJ Radosevich JL Hur EM The HMG-CoA reductase inhibitor, atorvastatin, promotes a Th2 bias and reverses paralysis in central nervous system autoimmune disease. Nature (2002) 420(6911):78–84. 10.1038/nature01158 12422218

[94] Aktas O Waiczies S Smorodchenko A Dorr J Seeger B Prozorovski T Treatment of relapsing paralysis in experimental encephalomyelitis by targeting Th1 cells through atorvastatin. J Exp Med (2003) 197(6):725–33. 10.1084/jem.20021425 12629065 PMC2193848

[95] Ifergan I Wosik K Cayrol R Kébir H Auger C Bernard M Statins reduce human blood-brain barrier permeability and restrict leukocyte migration: relevance to multiple sclerosis. Ann Neurol (2006) 60(1):45–55. 10.1002/ana.20875 16729291

[96] Society M Could Simvastatin Be a Treatment for Secondary Progressive MS. (2017). Available from: https://www.mssociety.org.uk/ms-stat2

[97] Greenwood J Steinman L Zamvil SS. Statin therapy and autoimmune disease: from protein prenylation to immunomodulation. Nat Rev Immunol (2006) 6(5):358–70. 10.1038/nri1839 16639429 PMC3842637

[98] Burridge K Wennerberg K. Rho and Rac take center stage. Cell (2004) 116(2):167–79. 10.1016/S0092-8674(04)00003-0 14744429

[99] Magee T Marshall C New insights into the interaction of Ras with the plasma membrane. Cell (1999) 98(1):9–12. 10.1016/S0092-8674(00)80601-7 10412976

[100] Tang JJ Li JG Qi W Qiu WW Li PS Li BL Inhibition of SREBP by a small molecule, betulin, improves hyperlipidemia and insulin resistance and reduces atherosclerotic plaques. Cell Metab (2011) 13(1):44–56. 10.1016/j.cmet.2010.12.004 21195348

[101] Daniel T Baráth M Benko S Széles L Dezso B Póliska S Activation of liver X receptor sensitizes human dendritic cells to inflammatory stimuli. J Immunol (2010) 184(10):5456–65. 10.4049/jimmunol.0902399 20410489

[102] Kirchgessner TG Sleph P Ostrowski J Lupisella J Ryan CS Liu X Beneficial and adverse effects of an LXR agonist on human lipid and lipoprotein metabolism and circulating neutrophils. Cell Metab (2016) 24(2):223–33. 10.1016/j.cmet.2016.07.016 27508871

[103] Lee SD Tontonoz P Liver X receptors at the intersection of lipid metabolism and atherogenesis. Atherosclerosis (2015) 242(1):29–36. 10.1016/j.atherosclerosis.2015.06.042 26164157 PMC4546914

[104] Lund EG Peterson LB Adams AD Lam MH Burton CA Chin J Different roles of liver X receptor alpha and beta in lipid metabolism: effects of an alpha-selective and a dual agonist in mice deficient in each subtype. Biochem Pharmacol (2006) 71(4):453–63. 10.1016/j.bcp.2005.11.004 16325781

[105] Quinet EM Savio DA Halpern AR Chen L Schuster GU Gustafsson JA Liver X receptor (LXR)-beta regulation in LXR alpha-deficient mice: implications for therapeutic targeting. Mol Pharmacol (2006) 70(4):1340–9. 10.1124/mol.106.022608 16825483

[106] Bovenga F Sabba C Moschetta A. Uncoupling nuclear receptor LXR and cholesterol metabolism in cancer. Cell Metab (2015) 21(4):517–26. 10.1016/j.cmet.2015.03.002 25863245

[107] Thangavel N Al Bratty M Akhtar Javed S Ahsan W Alhazmi HA Targeting peroxisome proliferator-activated receptors using thiazolidinediones: strategy for design of novel antidiabetic drugs. Int J Med Chem (2017):20 10.1155/2017/1069718 PMC5474549 28656106

[108] Kishimoto C Yuan Z Shioji K Peroxisome proliferation-activated receptor-gamma ligands ameliorate experimental autoimmune myocarditis. Circulation (2006) 114(18):624. 10.1016/s0008-6363(03)00457-7 14499870

[109] Zalba S ten Hagen TLM. Cell membrane modulation as adjuvant in cancer therapy. Cancer Treat Rev (2017) 52:48–57. 10.1016/j.ctrv.2016.10.008 27889637 PMC5195909

[110] Yang W Bai Y Xiong Y Zhang J Chen S Zheng X Potentiating the antitumour response of CD8+ T cells by modulating cholesterol metabolism. Nature (2016) 531(7596):651–5. 10.1038/nature17412 26982734 PMC4851431

[111] Kidani Y Bensinger SJ. Modulating cholesterol homeostasis to build a better T cell. Cell Metab (2016) 23(6):963–4. 10.1016/j.cmet.2016.05.015 27304495

